# Ultrasonic Irradiation Coupled with Microwave Treatment for Eco-friendly Process of Isolating Bacterial Cellulose Nanocrystals

**DOI:** 10.3390/nano8100859

**Published:** 2018-10-20

**Authors:** Endarto Yudo Wardhono, Hadi Wahyudi, Sri Agustina, François Oudet, Mekro Permana Pinem, Danièle Clausse, Khashayar Saleh, Erwann Guénin

**Affiliations:** 1 University of Sultan Ageng Tirtayasa, Cilegon 42435, Banten, Indonesia; hadi.wahyudi@untirta.ac.id (H.W); sri.agustina@hotmail.com (S.A); 2 Physico-Chemical Analysis Services, University of Technology of Compiègne, Sorbonne Universities, 60200 Compiègne, France; francois.oudet@utc.fr; 3 Integrated Transformations of Renewable Matter Laboratory (EA TIMR 4297 UTC-ESCOM), University of Technology of Compiègne, Sorbonne Universities, 60200 Compiègne, France; mekro-permana.pinem@utc.fr (M.P.P.); daniele.clausse@utc.fr (D.C.); khashayar.saleh@utc.fr (K.S.)

**Keywords:** bacterial cellulose nanocrystals, ultrasonic irradiation, microwave treatment, catalyzed hydrolysis, crystallinity index

## Abstract

The isolation of crystalline regions from fibers cellulose via the hydrolysis route generally requires corrosive chemicals, high-energy demands, and long reaction times, resulting in high economic costs and environmental impact. From this basis, this work seeks to develop environment-friendly processes for the production of Bacterial Cellulose Nanocrystals (BC-NC). To overcome the aforementioned issues, this study proposes a fast, highly-efficient and eco-friendly method for the isolation of cellulose nanocrystals from Bacterial Cellulose, BC. A two-step processes is considered: (1) partial depolymerization of Bacterial Cellulose (DP-BC) under ultrasonic conditions; (2) extraction of crystalline regions (BC-NC) by treatment with diluted HCl catalyzed by metal chlorides (MnCl_2_ and FeCl_3_.6H_2_O) under microwave irradiation. The effect of ultrasonic time and reactant and catalyst concentrations on the index crystallinity (CrI), chemical structure, thermal properties, and surface morphology of DP-BC and BC-NC were evaluated. The results indicated that the ultrasonic treatment induced depolymerization of BC characterized by an increase of the CrI. The microwave assisted by MnCl_2_-catalyzed mild acid hydrolysis enhanced the removal of the amorphous regions, yielding BC-NC. A chemical structure analysis demonstrated that the chemical structures of DP-BC and BC-NC remained unchanged after the ultrasonic treatment and MnCl_2_-catalyzed acid hydrolysis process.

## 1. Introduction

The most advantageous characteristics of the bio-based edible film are their edibility and inherent biodegradability [1]. Various biopolymers have been explored to reduce the use of non-degradable petroleum-based materials such as cellulose, chitosan, starch, collagen, pectin, etc. [2]. However, problems of strong hydrophilic character, high degradation, and inadequate mechanical properties in moist environments still limit the applications of biopolymers [3,4]. To become more applicable in practice, biopolymers have to be modified in terms of properties and functionalities [5]. In food packaging applications, for instance, the incorporation of reinforcement fillers [6,7] into the biopolymers matrix has shown to be an efficient strategy to overcome some critical issues [8] such as low mechanical resistance [9], hydrophilicity [10], and poor barrier to water vapor [11,12] compared to those of pure polymer or conventional (microscale) composites. More importantly, the process is less expensive compared to the development of new synthetic polymeric materials [13].

Nanocomposites represent an alternative to conventional technologies for improving biopolymer properties, by adding nanoparticles for which at least one dimension is in the nanometer range [14]. Most composite materials consist of one or more discontinuous phases distributed in one continuous phase. Discontinuous phase materials are usually harder and possess superior mechanical properties compared to continuous phase materials. The continuous phase is called the matrix, and the discontinuous one is called reinforcement [15]. The entity of the interactions is strongly affected by the nature of the discontinue phase; this can be maximized by passing from iso-dimensional particles to nanotubes [4]. Preparation of hybrid polymeric materials filled with natural particles also allows the fabrication of films with smart functions, such as antibacterial [16,17,18] and antioxidant capacities [19,20,21]. Numerous studies have been done on potential applications of biopolymers. Cellulose is an appropriate candidate used as a reinforcing material. Cellulose is a fibrous, tough, water-insoluble biomaterial that can play a substantial role in blending with different biopolymers to produce various bio-based nanocomposites [22]. Cellulose is the most abundant renewable biopolymer produced in the biosphere, and is obtained mainly from vegetables (plants and some algae species) and microbes (bacteria) [23].

Bacterial Cellulose, BC is constituted of fermented fibers, and is commonly synthesized by bacteria that are members of the Gluconacetobacter genus. Compared to cellulose plant fibers, BC displays higher crystallinity, and possesses improved properties such as high purity (with the absence of lignin and hemicellulose), ultrafine fibrous structure, low density, high water-retention capacity, and biocompatibility [24]. All these features make BC a promising biomaterial for industrial applications [25,26]. BC and plant fibers are both biopolymers that have similar molecular units but present a different structural organization. Depending on the source, plant fibers are mainly composed of three major components: cellulose, hemicellulose, and lignin. In contrast, the fibers made by bacteria are of pure cellulose; therefore, they present different physical properties [27]. Cellulose is a linear polysaccharide which consists of D-anhydro glucopyranose units linked by β-1,4-glycosidic bonds. The cellulose microfibrils have two types of structural regions: (i) the ordered region (crystalline) and (ii) the disordered region (amorphous). The crystalline regions give important mechanical properties to the cellulose fibers. Cellulose crystallinity, the degree of organization of the cellulose lattice, is a parameter describing the relative amount of crystalline content in the cellulose [28]. Crystallinity is a major factor affecting the activity of most celluloses; its values vary depending on the source and the mode of chemical treatment of the fibers [29].

Nanocrystals Cellulose (NCC) can be obtained by removing the amorphous regions while keeping the crystalline regions through partial depolymerization and purification from fiber sources. A comparison of the preparation of NCC from different natural materials and synthesis routes are presented in [30]. The most commonly-employed method to produce NCC is via acid hydrolysis conducted by strong mineral acids such as sulfuric acid, H_2_SO_4_, or hydrochloric acid, HCl [31]. The reaction involves the preferential hydrolysis of amorphous regions, promoting cleavage of glycosidic bonds. This procedure leads to the removal of the individual crystallites, which are regularly distributed along the microfibers, and drives to the formation of rod-like nanocrystals. The type of acid used determines the characteristics of the obtained NCC. H_2_SO_4_ will promote sulfonation of the crystallites surface [32] that produces a stable colloidal suspension due to electrostatic repulsion [33]. However, the presence of sulfate groups induces some crystallites to degrade, and reduces the thermostability of NCC [34,35,36]. It is generally known that low thermal stability may limit the use of nanocellulose and the manufacturing of its nanocomposites at high temperatures [37]. Although residual sulfate can be removed by dialysis, it is a time-consuming process, and particle aggregation is very difficult to avoid [38,39,40]. On the other hand, HCl produces hydroxyl groups on the surface of crystallites [41]. It generates a low-density surface charge with limited NCC dispersibility, which tends to promote flocculation in aqueous suspensions [42]. HCl is less corrosive than H_2_SO_4_, and though the yield is lower [41], it permits a significant increase in thermal stability of NCC [43]. To reach high yield value, a highly-concentrated aqueous solution of HCl is needed under hydrothermal conditions at 110 °C for a long period of the reaction [35].

As described in the previous paragraph, high-yield production of NCC is obtained using an excessive amount of mineral acids. Pollution to the environment, corrosion to the equipment, and the difficulty of controlling the reaction are the major limitations to synthesis using acid hydrolysis [44]. To overcome these issues, the aim of this study is to develop a fast, highly-efficient, and eco-friendly preparation method for the extraction of cellulose nanocrystals from Bacterial Cellulose, BC. A two-step process is considered, yielding Bacterial Cellulose NanoCrystals, BC-NC, namely: (1) partial depolymerization of BC under ultrasonic irradiation, (2) extraction of crystalline regions using microwave assisted by MnCl_2_-catalyzed hydrolysis. The effect of irradiation time on the partial depolymerization process and impact of MnCl_2_ concentration during the hydrolysis treatment is evaluated on the chemical structure, crystallinity index, thermal properties, and surface morphology of irradiated Depolymerized Bacterial Cellulose DP-BC and extracted BC-NC

## 2. Materials and Methods 

### 2.1. Materials

Nata de coco (BC pellicles) was collected from a local market, in Cilegon (Banten, Indonesia) region. Sodium hydroxide, NaOH, hydrochloric acid, HCl, and ethanol, C_2_H_5_OH were obtained from Thermo Fischer Scientific. Metal chlorides MnCl_2_, FeCl_3_.6H_2_O were purchased from Merck Indonesia (Jakarta, Indonesia). Commercial Microcrystal Cellulose, MCC was purchased from Sigma-Aldrich (Saint-Quentin, France) and Commercial Nanocrystal Cellulose, NCC from CelluForce, QC, Canada. All the reagents and chemicals are used as a laboratory grade without further purification. Demineralized water (conductivity of 0.06 mScm^−1^) produced by a purification chain was used for all experiments.

### 2.2. Methods 

#### 2.2.1. BC Preparation

BC pellicles were maintained in 0.5% NaOH (*w*/*v*) at room temperature for 24 h, followed by rinsing in the drained water until a neutral pH was attained; any chemicals used in the nata de coco production removed. The cellulose was then sun dried for two days, powdered, and sieved through a 149 µm sieve (100 Mesh).

#### 2.2.2. Partial Depolymerization of BC

The ultrasonic irradiation was carried out using an ultrasonic processor (Vibra Cell, Type 72434, 100 Watts, horn diameter: 1.0 mm, Fisher Scientific, Illkirch, France). One half of a gram (1% *w*/*v*) of BC powder was introduced into a 100-mL flat-bottom flask with a mixture of water/ethanol (50% *w*/*w*). The ultrasonic horn was placed at the center of the suspension, while the temperature was maintained at room temperature with a circulating water condenser. The suspension was constantly stirred at 300 rpm using magnetic stirring bar. All sonication runs were carried out at 20 kHz by varying irradiation times of 10, 20, 30, 60, and 120 min. After that, each sample of DP-BC was immediately washed with water and filtered with Whatman filter paper no. 1 until the filtrate was neutral. The DP-BC was then oven dried at 70 °C for 24 h.

#### 2.2.3. Extraction of Crystalline Regions

After ultrasonic irradiation treatment, the DP-BC (oven dry 0.3 g) was put into a microwave reaction vial (30 mL, G30), and 15 mL water of 0.1 mol/L HCl with a concentration of MnCl_2_ in the range 0; 1; 2.5; 5% *w*/*v* was added. The suspension was then placed into a microwave reactor (Anton Paar, Monowave 300) and heated as follows: (1) heating to 125 °C in 3 min; (2) maintaining the temperature at 125 °C for 30 min; and (3) cooling to 30 °C within 7 min. During the protocol, the suspension was stirred with a magnetic stirring bar at 1200 rpm. Upon completion of the hydrolysis, the vial was removed from the microwave oven and cooled at ambient temperature. The BC-NC suspension was transferred to a 50 mL plastic centrifuge tube and centrifuged at 12,000 rpm for 5 min (Jouan, MR 1812 Refrigerated Centrifuge, MN, USA) to remove residual acid and chemicals. The precipitate was purified by five washing cycles with deionized water followed by centrifugation at 12,000 rpm for 5 min. The BC-NC was then oven dried at 70 °C for 24 h before characterizations.

### 2.3. Characterization

#### 2.3.1. Fourier Transform Infrared (FT-IR)

FT-IR study was conducted to determine the functional groups present in the cellulose. The measurements were performed using a Nicolet iS5 spectrometer (Thermo Scientific, Whatman, MA, USA). Spectra were obtained between 4000 and 400 cm^−1^ at a resolution of 4 cm^−1^ and scanning speed of 20 mm/sat.

#### 2.3.2. X-Ray Diffraction (XRD)

XRD patterns of the cellulose were performed in a D8 Advance (Bruker, Bremen, Germany). Samples were examined with a scanning angle of 2θ from 10° to 40° at a rate of 1°/min with the CuKα filtered radiation. The crystallinity index, CrI was calculated from the diffraction intensity data using deconvolution method [45]. In which the diffraction profile was fitted by Gaussian function to find the contribution of each individual peak relative to the crystallographic planes and the amorphous background. The CrI was calculated Hermans equation as follows:

(1) $$\mathrm{CrI}=\left(\frac{A_{\mathrm{cr}}}{A_{\mathrm{cr}}{\;+\;A}_{\mathrm{am}}}\right)\times \;100\%$$

where A_am_ is the amorphous area, and A_cr_ is the sum of the area of the 101, 10ī, 002, 040 peaks.

#### 2.3.3. Differential Scanning Calorimetry (DSC)

DSC was carried out to analyze the thermal behavior of the cellulose. The samples were characterized on a DSC Q100 (TA Instruments, DE, USA) under constant nitrogen flow (50 mL/min), from 25 to 400 °C, at a heating rate of 10 °C/min.

#### 2.3.4. Transmission Electron Microscopy (TEM) and Scanning Transmission Electron Microscopy STEM)

The morphology of BC-NC suspension was measured by using the high-resolution JEOL-2100F TEM (Jeol, Akishima, Tokyo, Japan) in TEM and STEM mode. Samples were conventionally deposited on carbon coated copper grids and a negative staining was achieved using uranyLess solution (Delta Microscopies, Toulouse, France). The size and diameter distribution particle were measured by ImageJ (version 1.41 h) and origin pro-8 software.

## 3. Results

### 3.1. Chemical Structure

**Figure 1:**
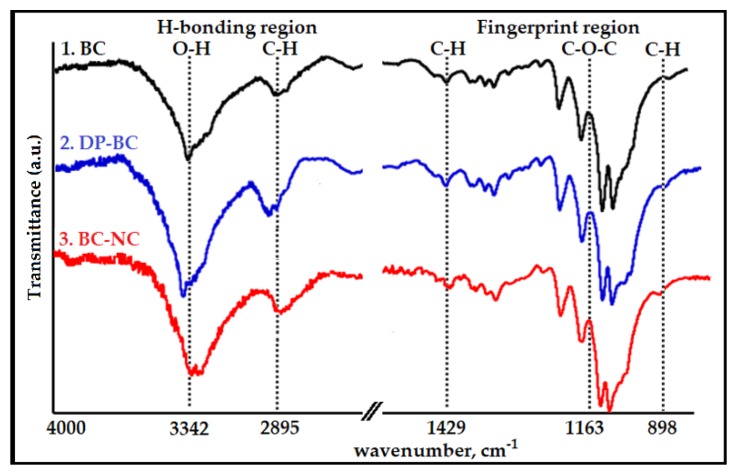
Fourier Transform Infrared (FT-IR) spectra of: 1. native Bacterial Cellulos (BC); 2. depolymerized cellulose, depolymerization of Bacterial Cellulose (DP-BC), (optimum conditions of ultrasonic irradiation step); 3. extracted crystalline regions, bacterial cellulose nanocrystals (BC-NC) (the best results of the catalyzed hydrolysis treatment).

FT-IR spectroscopy was used to investigate changes in the chemical structure of cellulose sample before and after the treatments. The spectra displayed the intensity of absorption of the functional groups between 4000 and 400 cm^−1^, which can identify the chemical bond in the cellulose molecule. The absorption bands for characteristic chemical groups of the raw material (native BC) and the treated celluloses (DP-BC and BC-NC) can be observed in Figure 1, and the typical vibration bands are listed in Table 1. The FT-IR spectra were divided into two parts: (1) H-bonding region from 4000 to 2600 cm^−1^, and (2) fingerprint region from 1800 to 400 cm^−1^ [46]. The broad peak in the 3650–3000 cm^−1^ bands was assigned to O–H stretching vibrations, which are characteristic of the hydroxyl groups generally present in cellulose, water, and lignin. In this region, intramolecular hydrogen bonds appeared at 3342 cm^−1^ and 3432 cm^−1^, and were attributed respectively to the two crystalline cellulose allomorphs, cellulose Iα and cellulose Iβ [47]. 

According to Börjesson and Westman [48], these hydroxyl groups were responsible for the stiffness in the polymer chain, and for allowing the linear polymers to form sheet structures. The strong vibration band around 2895 cm^−1^ corresponded to C–H stretching vibrations [49]. This band may be associated with a hydrocarbonate linear chain. Higher values in this specific band are correlated to a decrease in the calculated total crystallinity value [50]. An intense band at 1429 cm^−1^ band can be assigned to the bending of asymmetric angular deformation of C–H bonds. The band found between 1420 to 1430 cm^−1^ was associated with the amount of the cellulose ordered form, while the band appearing at 898 cm^−1^ was assigned to the disordered region [51]. The 1163 cm^−1^ band was assigned to asymmetrical stretching of C–O–C glycoside bonds.

### 3.2. Crystallinity Index

**Figure 2:**
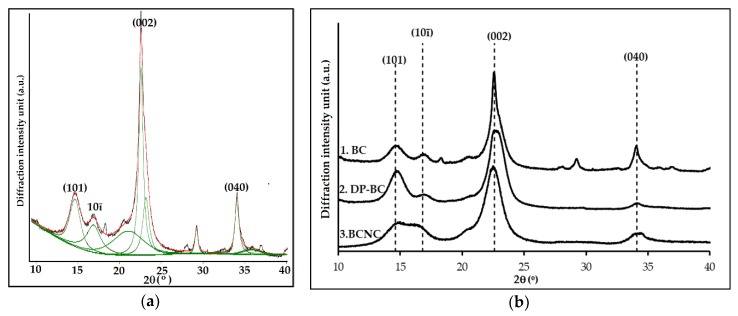
X-ray diffractogram of: (**a**) BC that was fitted by Gauss function; (**b**) XRD pattern of: 1. native BC; 2. depolymerized cellulose, DP-BC, (the optimum result of ultrasonic irradiation step); 3. extracted crystalline regions, BC-NC (the best results of the catalyzed hydrolysis treatment).

The crystallinity index of the cellulose was analyzed by X-ray Diffraction analysis. The CrI was calculated by curve-fitting process where individual crystalline peaks were extracted from the diffraction intensity profiles [52,53]. X-ray diffractogram of BC sample was fitted by Gauss function; the results are shown in Figure 2a, and the optimum results of the partial depolymerization step and the extraction of crystalline domains step are presented in Figure 2b. The integrated peak area obtained of each fitting curve is shown in Table 2. 

There were at least eight peaks that had been separated from the diffraction intensity profiles but only four distinct characteristic peaks at 2θ = 14.6°, 16.8°, 22.6°, and 34.1°, which were considered to correspond to 101, 10ī, 002, and 040 crystallographic planes [54,55]. The broad peaks were attributed to the amorphous contribution. The assumption for this analysis was that the amorphous contribution increase was the main contributor to peak broadening. According to Park et al. [45], the other intrinsic factors that influence peak broadening were crystallite size and non-uniform strain within the crystal. These assumptions were then utilized to carry out an investigation of the crystallinity for all the samples. After subtracting the amorphous regions from the whole samples, the CrI was calculated by dividing the remaining diffractogram area due to crystalline cellulose by the total area of the original diffractogram. The samples presented correspond to the optimum results of the partial depolymerization step (DP-BC) which have been further hydrolyzed into the microwave reactor (BC-NC).

### 3.3. Thermal Properties

**Figure 3:**
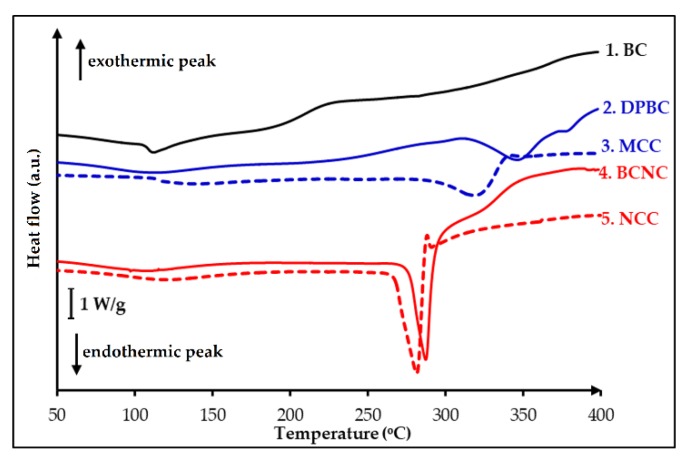
Differential Scanning Calorimetry (DSC)-Thermograms of: 1. BC; 2. depolymerized cellulose, DP-BC; 3. extracted nanocrystalline cellulose, BC-NC.

Crystallinity is an important parameter, which can greatly affect the physical properties of biodegradable polymers. The identification of neat polymers, copolymers, polymer blends, and composites, as well as the determination of their purity and stability, are generally described by DSC. Amorphous polymers exhibit a glass transition temperature while crystalline or semi-crystalline polymers may possess glass transition temperature, a freezing and melting temperature with various freezing, and melting enthalpies [56]. In this work, the glass transition temperature, T_g_, melting point, T_m_, and decomposition temperature, T_d_ were investigated to interpret thermal behaviors of the extracted celluloses. The DSC thermograms for the BC, DP-BC, BC-NC and the reference materials, commercial Microcrystal Cellulose, MCC and Nanocrystal Cellulose, NCC were registered at a heating rate of 10 °C/min and depicted in Figure 3. The thermograms revealed that BC exhibited different pattern from DP-BC and BC-NC. The heat-flow curve of BC displayed a small inflection of the baseline around 105–110 °C, which is the glass transition temperature, T_g_, and is followed by an endothermic peak with the onset, T_m_ = 113.8 °C. In the treated celluloses, DP-BC and BC-NC, with the increased in crystalline content (CrI), the endothermic peak shifted toward a higher temperature. This slightly marked peak could be attributed to the presence of an amorphous region [57]. The curve of DP-BC showed an endothermic peak at 310–370 °C, which appeared to be a melting temperature at T_m_ = 348.7 °C. The peak was followed by a degradation temperature at T_d_ = 381.3 °C of the cellulosic material. A similar result was found for BC-NC: an endothermic peak was detected around 260–290 °C with the onset temperature at T_m_ = 282.8 °C then followed by degradation at T_d_ = 318.6 °C. The T_g_ of both samples, DP-BC and BC-NC disappeared or became difficult to detect because of the partial removal of the amorphous regions.

### 3.4. Morphology

**Figure 4:**
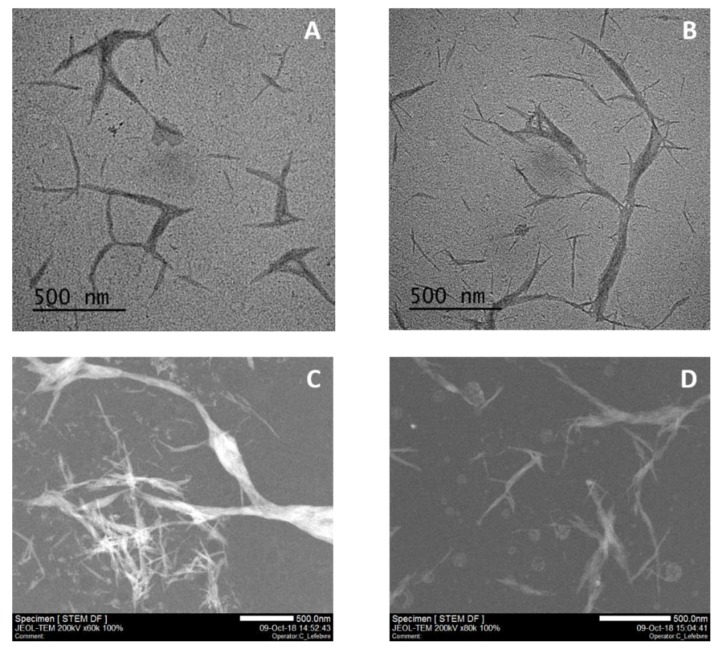
Transmission Electron Microscopy (TEM) and Scanning Transmission Electron Microscopy (STEM) micrograph of BC-NC (**A**,**C**) produced with 0.1M HCl and 5% *w*/*w* of MnCl_2_ compare to commercial NCC (**B**,**D**).

The morphology of nanocrystals after the hydrolysis treatment was characterized by TEM and STEM observations. Figure 4 presents a comparison of TEM and STEM micrograph of sample BC-NC obtained from a 30 min microwave reaction with MnCl_2_ catalyst (5% *w*/*w*) at 125 °C to commercial NCC. Both samples were prepared in the same conditions, and as shown, are similarly constituted of a mixture of fibrillated structure with variable length and smaller needles or nano-rods. It seems that the fibrillated structures are in fact constituted of the densely-packed needles. The BC-NC nano-rods are of (164.51 ± 7.56) nm in length with an average diameter of (25.05 ± 2.80) nm. They look like the commercial forms which are (90.94 ± 10.05) nm in length with an average diameter of (12.58 ± 0.87) nm. This result confirmed the crystalline structure already described with a very close similarity to commercially-available nanocrystalline cellulose. 

## 4. Discussion 

### 4.1. Effect of Ultrasonic Irradiation on The Depolymerization Cellulose

The term “ultrasonic” describes sound waves with a frequency greater than 20 kHz. Many studies have reported the exposure to this wave is responsible for a number of physical and chemical changes. The ultrasonication was the adopted method here to carry out partial depolymerization of native BC into microfibers. The utilization of ultrasonic waves offers a simple and versatile tool for synthesizing micro or nanostructured materials that are often unavailable by conventional methods. In this work, native BC was irradiated into a mixture of water/ethanol (50% *w*/*w*) at constant power of 100 W and frequency of 20 kHz by varying irradiation times. The influence of the length of ultrasonic irradiation period on the CrI is presented in Table 3.

From the table, it could be concluded that the increase of CrI is dependent on the irradiation time. Ultrasonic irradiation in water/ethanol induced partial depolymerization of BC with a CrI increase of 8.4% during the first 30 min. The maximum CrI was observed to be 71.4% at 120 min. The results indicated that the irradiation leads to the rupture of amorphous cellulose chains. The disintegration of amorphous regions may be explained by acoustic cavitation. As native BC in a liquid medium was exposed to ultrasonic irradiation, the acoustic waves induce alternating high and low pressure; this creates bubbles (i.e., cavities) and makes them oscillate. A bubble can grow while absorbing the ultrasonic energy at each cycle, until it becomes unstable and finally collapses violently, releasing the energy stored within it, subsequently producing shock waves in the medium [58]. A shear deformation during the collapse of the bubbles is considered to be responsible for the chemical effects which induce disintegration of the amorphous regions of cellulose.

**Figure 5:**
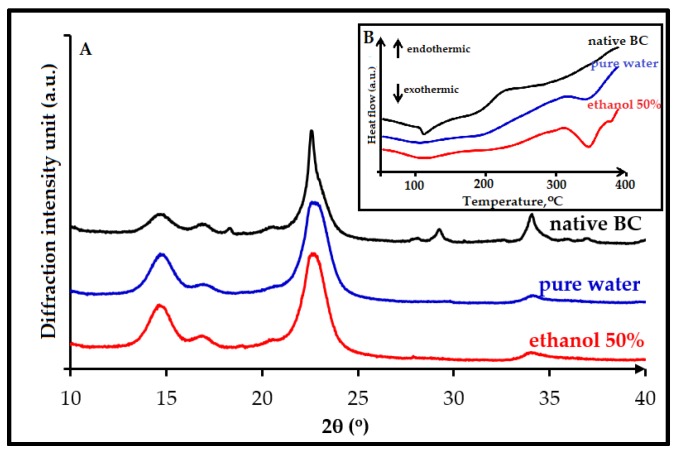
The evolutions of crystalline regions formations for treatment of bacterial cellulose after 30 min irradiation time in pure water (blue curve) or a mixture of ethanol / water 50% (*w*/*w*) (red line) observed by XRD (**A**) and by DSC (**B** in insert) (untreated BC, dark line).

Cavitation occurs over a very wide range of frequencies, from 10 Hz to 10 MHz. Above that frequency regime, the intrinsic viscosity of liquids prevents cavitation from occurring. According to Suslick and coworkers [59], most high intensity ultrasonic horns operate within the range of 20 to 40 kHz. Several factors can affect acoustic cavitation, such as reaction temperature, hydrostatic pressure, frequency, acoustic power, and the type of the solvent medium used. In our study, the amorphous regions degradation increases slightly after 30 min irradiation, and the CrI of cellulose for all sample experiments is still lower compared with that of commercial MCC (75.0%). In our experiments, optimum crystallinity is observed with 30 min irradiation; then, when reaction time is increased, degradation might occurred in both the amorphous and crystalline regions, consecutively reducing the product crystallinity. Concerning the solvent medium, when more volatile solvent is used such as ethanol in water, the mixture is expected to produce more cavitation bubbles, which can significantly promote the reduction rate of amorphous regions. We therefore compare our results (using water/ethanol 50% *w*/*w*) to reactions done in pure water. From XRD spectra, it was calculated that the increase in CrI was less pronounced when the reaction was run in sole water (increase from 60.7 to 67.0%) than in water/ethanol mixture (increase from 60.7 to 69.1%). This result is in accordance with DSC observations, as the endothermic peak of sample that was irradiated with ethanol (the red line in Figure 5B) was detected broader than the one with pure water (blue line in Figure 5B). Both samples have the same melting temperature, i.e., around T_m_ = 344 °C. The endothermic peak for sample as detected in the DSC curves becomes larger when the crystallinity of the sample increases. According to Ciolacu et al., 2011 [57], the broadening of endothermic peaks detected in the DSC curves of celluloses is in a linear relationship with the percentage value of the amorphous material from their crystalline structure.

### 4.2. Effect of MnCl_2_ Concentration on The Extraction of Crystalline Regions

The microwave treatment assisted by HCl-MnCl_2_ catalyzed hydrolysis was evaluated to hydrolyze cellulose. In this part, the use of a microwave reactor was performed to get a higher conversion and a shorter reaction time for catalyzed hydrolysis of depolymerized cellulose. In comparison, a conventional heating microwave is a high-frequency radiation that possesses both electrical and magnetic properties [60]. Regarding the addition of catalysts, MnCl_2_, was utilized to improve the extraction rate of crystalline regions during the hydrolysis. It was already shown that metal chlorides, due to their Lewis acid property, exhibit higher catalytic activity than inorganic acids [61]. The concentration of HCl used in this work was significantly reduced to 0.1 M, instead of 6 M, as used in many reported works.

Table 4 shows the effect of the metal chlorides catalyzed hydrolysis reaction on the crystallinity index. In the absence of a catalyst (0%) in the 0.1M HCl medium, thermal hydrolysis could not occur effectively, and the CrI obtained was only 0.5% higher than for the starting material (DP-BC) from 69.1% to 69.6% for 30 min of reaction. Conversely, with the addition of MnCl_2_, the CrI was increased to 71.3%, 72.7%, and 79.4% for 1%, 2.5%, and 5% (*w*/*w*), respectively. A similar result was obtained for the use of FeCl_3_·6H_2_O, 5% (*w*/*w*) as catalyst with a CrI increased from 69.1% to 77.8%. It was also found that the CrI of all experiments showed a lower value compared with that of the commercial NCC (85.0%). Nevertheless, it appears that the presence of a catalyst plays an important role in the extraction of crystalline regions. In our hypothesis, during partial depolymerization, the ultrasonic treatment leads to the distortion of the amorphous parts and eases the accessibility of chemical reagent to loosen them. Thus, the protons could more easily penetrate into the disordered regions during catalyzed hydrolysis, and as a result, greatly promote the hydrolytic cleavage of glycosidic bonds even in the diluted HCl medium. For this step, the hydrolysis reaction at 0.1M of HCl and 5% *w*/*w* of both metal chlorides (MnCl_2_ and FeCl_3_·6H_2_O) for 30 min reaction can enhance the removal of the amorphous regions, even though the CrI obtained is still less than 80%.

The rapid degradation of amorphous regions during catalyzed hydrolysis can be explained by the Lewis acid character. According to Stein et al., 2010 [62], some metal chlorides such as FeCl_3_, AlCl_3_, CuCl_2_, and MnCl_2_ could form hydrated complexes in aqueous solution and coordinate the glycosidic oxygen of cellulose. This helps to scissor the glycosidic linkages and to facilitate the hydrolysis process, while the chloride anions attack the hydroxyl atoms [61,63,64]. Introducing metal chloride salts into acid solution can further improve catalytic performance at which the intra- and inter-molecular hydrogen bonds can be broken, and the degradation of the amorphous regions can be accelerated by permeating the internal structure of irradiated cellulose (DP-BC) to acid. Moreover, under microwave and hydrothermal conditions, the easy diffusion of metal cations and chloride anions into the hydrogen bond network, as well as the strong ability of chloride anions to disrupt the hydrogen bond, can be achieved; thus, the hydrolysis rate is greatly enhanced.

Considering the thermal behavior analysis, the summary of DSC results is presented in Table 5 as follows:

It was found that compared with the DP-BC, the degradation temperature of the BC-NC decreased by approximately 62.7 °C. Similar results were obtained for commercial MCC and NCC sample references. The nano-sized NCC exhibited lower degradation temperature than the micro-sized MCC, i.e., by 53.7 °C. The reason is that the thermal stability of nanocrystals is related to several factors including their dimension, crystallinity, and composition, which in turn depend on extraction conditions [65,66]. So, the NCC with the highest crystallinity would exhibit the highest thermal stability, but smaller dimensions should also cause a decrease of the degradation temperature. The FT-IR analysis demonstrated that the chemical structures of BC-NC remained unchanged after MnCl_2_-catalyzed hydrolysis process.

## 5. Conclusions

In this study, BC-NC was conveniently synthesized by sequential ultrasonic irradiation and microwave treatment. A simple and an eco-friendly approach was developed to control the degradation of bacterial cellulose at very low hydrochloric acid concentration. The results demonstrated that ultrasonic irradiation of BC in water/ethanol mixture led to cellulose depolymerization. The CrI increased from 60.7% to 69.1% during the first 30 min of irradiation. Yet, amorphous region distortion remained constant or increased slightly after 2 h, i.e., by 71.4%. It was found that microwave treatment using MnCl_2_ as Lewis acid exhibited excellent catalytic activity and promoted the hydrolysis in diluted HCl. The reaction rate and the selectivity of BC-NC formation are shown to be optimal at 0.1 M of HCl with 5% *w*/*v* of MnCl_2_ for 30 min. These conditions can enhance the removal of amorphous regions, yielding BC-NC that possesses an initiating decomposition temperature of 318.6 °C, and led to improve the CrI of up to 79.4%. The BC-NC is typically a mixture of small needles of (164.51 ± 7.56) nm in length and (25.05 ± 2.80) nm diameter that can form fibrillated structures. This procedure yields nanocrystalline bacterial cellulose having similar features to commercially-available nanocrystalline cellulose.

## Figures and Tables

**Table 1:** Typical vibration bands for the Fourier Transform Infrared (FTIR) spectra of the cellulose samples Bacterial Cellulos (BC), depolymerization of Bacterial Cellulose (DP-BC) and bacterial cellulose nanocrystals (BC-NC).

Sample	Wavenumber, cm^−1^				
	H-bonding region		Fingerprint Print Region		
	Stretching of O–H bonds	Symmetric C–H stretching vibration	Asymmetric Angular Deformation of C–H (Crystalline Regions)	Asymmetrical C–O–C Glycoside Bonds	Asymmetric Angular Deformation of C–H (Amorphous Region)
BC					
DP-BC	3342	2895	1429	1163	898
BC-NC					

**Table 2:** The integrated peak area obtained of each fitting curve.

Sample	Area (2θ)							
	14.6°	16.8°	22.6°	23.8°	27.2°	29.2°	34.1°	34.8°
	(101)	(10ī)	(002)				(040)	
BC	41.8	46.8	324.0	88.3	27.9	125.9	37.6	48.9
DP-BC	95.8	36.5	583.8	26.1	23.0	10.1	33.5	276.6
BC-NC	46.8	38.1	420.6	110.8	17.8	51.9	122	73.6

**Table 3:** Influence of the length of ultrasonic irradiation period on Crystallinity Index, CrI.

Sample	Integrated Area						
	BC	Length of Ultrasonic Irradiation Period (min)					
		10	20	30	60	120	MCC ^1^
A_cr_	450.2	665.3	700.3	749.4	692.6	586.3	637.5
A_tot_	741.2	1016.0	1032.9	1085.2	979.4	821.1	850.5
CrI	60.7%	65.5%	67.8%	69.1%	70.7%	71.4%	75.0%

^1^ Microcrystal Cellulose, MCC (commercial).

**Table 4:** Influence of concentration of catalyst MnCl_2_ and FeCl_3_·6H_2_O on Crystallinity Index, CrI.

Integrated Area							
Catalyst Concentration (% *w*/*w*)							
	DP-BC	MnCl_2_				FeCl_3_·6H_2_O	NCC ^2^
		0	1	2.5	5		
A_cr_	749.4	570.4	550.8	508.4	700.2	581.3	1068.1
A_am_	335.2	249.3	221.7	191.0	181.4	747.2	186.6
^CrI^	69.1%	69.6%	71.3%	72.7%	79.4%	77.8%	85.1%

^2^ NanoCrystal Cellulose, NCC (commercial).

**Table 5:** Characteristic thermal behavior of sample celluloses.

Temperature	Sample ( °C)				
	BC	DP-BC	MCC	BC-NC	NCC
T_g_	105.0	-	-	-	-
T_m_	113.8	348.7	314.1	282.8	278.7
T_d_	160.7	381.3	340.1	318.6	286.4

## References

[1] Guilbert S., Gontard N., Gorris L.G. (1996). Prolongation of the shelf-life of perishable food products using biodegradable films and coatings. LWT-Food Sci. Technol..

[2] Makaremi M., Pasbakhsh P., Cavallaro G., Lazzara G., Aw Y.K., Lee S.M., Milioto S. (2017). Effect of morphology and size of halloysite nanotubes on functional pectin bionanocomposites for food packaging applications. ACS Appl. Mater. Interfaces.

[3] Khan A., Khan R.A., Salmieri S., Tien C.L., Riedl B., Bouchard J., Chauve G., Tan V., Kamal M.R., Lacroix M. (2012). Mechanical and barrier properties of nanocrystalline cellulose reinforced chitosan based nanocomposite films. Carbohydr. Polym..

[4] Bertolino V., Cavallaro G., Lazzara G., Merli M., Milioto S., Parisi F., Sciascia L. (2016). Effect of the biopolymer charge and the nanoclay morphology on nanocomposite materials. Ind. Eng. Chem. Res..

[5] Khan A., Huq T., Saha M., Khan R.A., Khan M.A., Gafur M.A. (2010). Effect of silane treatment on the mechanical and interfacial properties of calcium alginate fiber reinforced polypropylene composite. J. Compos. Mater..

[6] De Silva R.T., Pasbakhsh P., Goh K.L., Chai S.P., Chen J. (2014). Synthesis and characterisation of poly (lactic acid)/halloysite bionanocomposite films. J. Compos. Mater..

[7] Gorrasi G., Pantani R., Murariu M., Dubois P. (2014). PLA/H alloysite Nanocomposite Films: Water Vapor Barrier Properties and Specific Key Characteristics. Macromol. Mater. Eng..

[8] Cataldo V.A., Cavallaro G., Lazzara G., Milioto S., Parisi F. (2017). Coffee grounds as filler for pectin: Green composites with competitive performances dependent on the UV irradiation. Carbohydr. Polym..

[9] Gorrasi G., Bugatti V., Vittoria V. (2012). Pectins filled with LDH-antimicrobial molecules: Preparation, characterization and physical properties. Carbohydr. Polym..

[10] Cavallaro G., Donato D.I., Lazzara G., Milioto S. (2011). Films of halloysite nanotubes sandwiched between two layers of biopolymer: From the morphology to the dielectric, thermal, transparency, and wettability properties. J. Phys. Chem. C..

[11] Lagaron J.M., Lopez-Rubio A. (2011). Nanotechnology for bioplastics: Opportunities, challenges and strategies. Trends Food Sci. Technol..

[12] Pantani R., Gorrasi G., Vigliotta G., Murariu M., Dubois P. (2013). PLA-ZnO nanocomposite films: Water vapor barrier properties and specific end-use characteristics. Eur. Polym. J..

[13] Vroman I., Tighzert L. (2009). Biodegradable polymers. Materials..

[14] Ruiz-Hitzky E., Aranda P., Darder M., Rytwo G. (2010). Hybrid materials based on clays for environmental and biomedical applications. J. Mater. Chem..

[15] Berthelot J.M. (2012). Composite materials: Mechanical behavior and structural analysis.

[16] Lvov Y., Wang W., Zhang L., Fakhrullin W. (2016). Halloysite clay nanotubes for loading and sustained release of functional compounds. Adv. Mater..

[17] Abdullayev E., Lvov Y. (2013). Halloysite clay nanotubes as a ceramic ‘skeleton’ for functional biopolymer composites with sustained drug release. J. Mater. Chem. B.

[18] Abdullayev W., Sakakibara K., Okamoto K., Wei W., Ariga K., Lvov Y. (2011). Natural tubule clay template synthesis of silver nanorods for antibacterial composite coating. ACS Appl. Mater. Interfaces.

[19] Biddeci G., Cavallaro G., Di Blasi F., Lazzara G., Massaro M., Milioto S., Parisi F., Riela S., Spinellia G. (2016). Halloysite nanotubes loaded with peppermint essential oil as filler for functional biopolymer film. Carbohydr. Polym..

[20] Gorrasi G. (2015). Dispersion of halloysite loaded with natural antimicrobials into pectins: Characterization and controlled release analysis. Carbohydr. Polym..

[21] Massaro M., Riela S., Guernelli S., Parisi F., Lazzara G., Baschieri A., Valgimigli L., Amorati R. (2016). A synergic nanoantioxidant based on covalently modified halloysite–trolox nanotubes with intra-lumen loaded quercetin. J. Mater. Chem. B.

[22] Abdulkhani A., Marvast E.H., Ashori A., Hamzeh Y., Karimi A.N. (2013). Preparation of cellulose/polyvinyl alcohol biocomposite films using 1-n-butyl-3-methylimidazolium chloride. Int. J. Biol. Macromol..

[23] Qiu X., Hu S. (2013). Smart’ materials based on cellulose: A review of the preparations, properties, and applications. Materials.

[24] Paximada P., Tsouko E., Kopsahelis N., Koutinas A. A., and Mandala I. (2016). Bacterial cellulose as stabilizer of o/w emulsions. Food Hydrocoll..

[25] Hu Y., Catchmark J.M., Zhu Y., Abidi N., Zhou X., Wang J., Liang H. (2014). Engineering of porous bacterial cellulose toward human fibroblasts ingrowth for tissue engineering. J. Mater. Res..

[26] Shah N., Ul-Islam M., Khattak W. A., and Park J. K. (2013). Overview of bacterial cellulose composites: A multipurpose advanced material. Carbohydr. Polym..

[27] Brown A.J. (1886). XLIII-On an acetic ferment which forms cellulose. J. Chem. Soc. Trans..

[28] Li J., Zhang X., Zhang M., Xiu H., and He H. (2014). Optimization of selective acid hydrolysis of cellulose for microcrystalline cellulose using FeCl_3_. BioResources..

[29] George J., Ramana K.V., Sabapathy S.N., Jagannath J.H., Bawa A.S. (2005). Characterization of chemically treated bacterial (*Acetobacter xylinum*) biopolymer: Some thermo-mechanical properties. Int. J. Biol. Macromol..

[30] Thambiraj S., Shankaran D.R. (2017). Preparation and physicochemical characterization of cellulose nanocrystals from industrial waste cotton. Appl. Surf. Sci..

[31] Dufresne A. (2012). Nanocellulose: From Nature to High Performance Tailored Materials.

[32] Ureña-Benavides E.E., Davis G., Ao V.A., Kitchens C.L. (2011). Rheology and phase behavior of lyotropic cellulose nanocrystal suspensions. Macromolecules..

[33] Beck-Candanedo S., Roman M., Gray D.G. (2005). Effect of reaction conditions on the properties and behavior of wood cellulose nanocrystal suspensions. Biomacromolecules.

[34] Reid M.S., Villalobos M., Cranston E.D. (2016). Benchmarking cellulose nanocrystals: From the laboratory to industrial production. Langmuir.

[35] Yu H., Qin Z., Liang B., Liu N., Zhou Z., Chen L. (2013). Facile extraction of thermally stable cellulose nanocrystals with a high yield of 93% through hydrochloric acid hydrolysis under hydrothermal conditions. J. Mater. Chem. A.

[36] Roman M., Winter W.T. (2004). Effect of sulfate groups from sulfuric acid hydrolysis on the thermal degradation behavior of bacterial cellulose. Biomacromolecules.

[37] Sheltami R.M., Kargarzadeh H., Abdullah I., Ahmad I. (2017). Thermal Properties of Cellulose Nanocomposites. Handbook of Nanocellulose and Cellulose Nanocomposites.

[38] Filson P.B., Dawson-Andoh B.E. (2009). Sono-chemical preparation of cellulose nanocrystals from lignocellulose derived materials. Bioresour. Technol..

[39] Jiang F., Esker A.R., Roman M. (2010). Acid-catalyzed and solvolytic desulfation of H_2_SO_4_-hydrolyzed cellulose nanocrystals. Langmuir.

[40] Rosa M.F., Medeiros E.S., Malmonge J.A., Gregorski K.S., Wood D.F., Mattoso L.H.C., Glenn G., Orts W.J., Imam S.H. (2010). Cellulose nanowhiskers from coconut husk fibers: Effect of preparation conditions on their thermal and morphological behavior. Carbohydr. Polym..

[41] Araki J., Wada M., Kuga S., Okano T. (1998). Flow properties of microcrystalline cellulose suspension prepared by acid treatment of native cellulose. Colloids Surf. Physicochem. Eng. Asp..

[42] Martínez-Sanz M., Lopez-Rubio A., Lagaron J.M. (2011). Optimization of the nanofabrication by acid hydrolysis of bacterial cellulose nanowhiskers. Carbohydr. Polym..

[43] Yu H.Y., Qin Z.Y., Liu L., Yang X.G., Zhou Y., Yao J.M. (2013). Comparison of the reinforcing effects for cellulose nanocrystals obtained by sulfuric and hydrochloric acid hydrolysis on the mechanical and thermal properties of bacterial polyester. Compos. Sci. Technol..

[44] Brinchi L., Cotana F., Fortunati E., Kenny J.M. (2013). Production of nanocrystalline cellulose from lignocellulosic biomass: Technology and applications. Carbohydr. Polym..

[45] Park S., Baker J.O., Himmel M.E., Parilla P.A., Johnson D.K. (2010). Cellulose crystallinity index: Measurement techniques and their impact on interpreting cellulase performance. Biotechnol. Biofuels.

[46] Kruer-Zerhusen N., Cantero-Tubilla B., Wilson D. B. (2018). Characterization of cellulose crystallinity after enzymatic treatment using Fourier transform infrared spectroscopy (FTIR). Cellulose.

[47] Kondo T. (1997). The assignment of IR absorption bands due to free hydroxyl groups in cellulose. Cellulose.

[48] Börjesson M., Westman G. (2015). Crystalline nanocellulose: Preparation, modification, and properties. Cellulose-Fundamental Aspects and Current Trends.

[49] Chang W.S., and Chen H.H. (2016). Physical properties of bacterial cellulose composites for wound dressings. Food Hydrocoll..

[50] Ornaghi H.L., Poletto M., Zattera A.J., Amico S.J. (2014). Correlation of the thermal stability and the decomposition kinetics of six different vegetal fibers. Cellulose.

[51] AAkerholm M., Hinterstoisser B., Salmén L. (2004). Characterization of the crystalline structure of cellulose using static and dynamic FT-IR spectroscopy. Carbohydr. Res..

[52] Hult E.L., Iversen T., Sugiyama J. (2003). Characterization of the supermolecular structure of cellulose in wood pulp fibres. Cellulose.

[53] Garvey C.J., Parker I.H., and Simon G.P. (2005). On the interpretation of X-ray diffraction powder patterns in terms of the nanostructure of cellulose I fibres. Macromol. Chem. Phys..

[54] He J., Cui S., Wang S. (2008). Preparation and crystalline analysis of high-grade bamboo dissolving pulp for cellulose acetate. J. Appl. Polym. Sci..

[55] Nishiyama Y., Sugiyama J., Chanzy H., Langan P. (2003). Crystal structure and hydrogen bonding system in cellulose Iα from synchrotron X-ray and neutron fiber diffraction. J. Am. Chem. Soc..

[56] Gregorova A. (2013). Application of differential scanning calorimetry to the characterization of biopolymers. Applications of Calorimetry in a Wide Context-Differential Scanning Calorimetry, Isothermal Titration Calorimetry and Microcalorimetry, Odile Carisse.

[57] Ciolacu D., Ciolacu F., Popa V. I. (2011). Amorphous cellulose—Structure and characterization. Cellul. Chem. Technol..

[58] Suslick K.S. (1990). Effects of ultrasound on surfaces and solids. Adv. Sonochem..

[59] Bang J. H., and Suslick K. S. (2010). Applications of ultrasound to the synthesis of nanostructured materials. Adv. Mater..

[60] Wong T.W., Chan L.W., Kho S.B. (2002). Heng, P.W.S. Design of controlled-release solid dosage forms of alginate and chitosan using microwave. J. Control. Release..

[61] Kamireddy S.R., Li J., Tucker M., Degenstein J., Ji Y. (2013). Effects and mechanism of metal chloride salts on pretreatment and enzymatic digestibility of corn stover. Ind. Eng. Chem. Res..

[62] vom Stein T., Grande P., Sibilla F., Commandeur U., Fischer R., Leitner W., Domínguez de María P. (2010). Salt-assisted organic-acid-catalyzed depolymerization of cellulose. Green Chem..

[63] Lu Q., Tang L., Lin F., Wang S., Chen Y., Chen X., Huang B. (2014). Preparation and characterization of cellulose nanocrystals via ultrasonication-assisted FeCl_3_-catalyzed hydrolysis. Cellulose.

[64] Ma Y., Ji W., Zhu X., Tian L., Wan X. (2012). Effect of extremely low AlCl_3_ on hydrolysis of cellulose in high temperature liquid water. Biomass Bioenergy.

[65] Henrique M.A., Neto W.P.F., Silvério H.A., Martins D.F., Gurgel L.V.A., Barud H.S., Morais L.C., Pasquini D. (2015). Kinetic study of the thermal decomposition of cellulose nanocrystals with different polymorphs, cellulose I and II, extracted from different sources and using different types of acids. Ind. Crops Prod..

[66] Jonoobi M., Oladi R., Davoudpour Y., Oksman K., Dufresne A., Hamzeh Y., Davoodi R. (2015). Different preparation methods and properties of nanostructured cellulose from various natural resources and residues: A review. Cellulose.

